# Estrogen receptors in granulosa cells govern meiotic resumption of pre-ovulatory oocytes in mammals

**DOI:** 10.1038/cddis.2017.82

**Published:** 2017-03-09

**Authors:** Wei Liu, Qiliang Xin, Xiao Wang, Sheng Wang, Huarong Wang, Wenqiang Zhang, Ye Yang, Yanhao Zhang, Zhiyuan Zhang, Chao Wang, Yang Xu, Enkui Duan, Guoliang Xia

**Affiliations:** 1 State Key Laboratory of Agrobiotechnology, College of Biological Sciences, China Agricultural University, No. 2 Yuanmingyuan West Road, Beijing 100193, China; 2 Department of Gynecology and Obstetrics, Medical Center of Reproduction and Genetics, Peking University First Hospital, Beijing 100034, China; 3 College of Biotechnology, Tianjin University of Science and Technology, Tianjin 300457, China; 4 State Key Laboratory of Stem Cell and Reproductive Biology, Institute of Zoology, Chinese Academy of Sciences, Beijing 100101, China

## Abstract

In mammals, oocytes are arrested at the diplotene stage of meiosis I until the pre-ovulatory luteinizing hormone (LH) surge triggers meiotic resumption through the signals in follicular granulosa cells. In this study, we show that the estradiol (E2)-estrogen receptors (ERs) system in follicular granulosa cells has a dominant role in controlling oocyte meiotic resumption in mammals. We found that the expression of ERs was controlled by gonadotropins under physiological conditions. E2-ERs system was functional in maintaining oocyte meiotic arrest by regulating the expression of natriuretic peptide C and natriuretic peptide receptor 2 (NPPC/NPR2), which was achieved through binding to the promoter regions of *Nppc* and *Npr2* genes directly. In ER knockout mice, meiotic arrest was not sustained by E2 in most cumulus–oocyte complexes *in vitro* and meiosis resumed precociously in pre-ovulatory follicles *in vivo*. In human granulosa cells, similar conclusions are reached that ER levels were controlled by gonadotropins and E2-ERs regulated the expression of NPPC/NPR2 levels. In addition, our results revealed that the different regulating patterns of follicle-stimulating hormone and LH on ER levels *in vivo versus in vitro* determined their distinct actions on oocyte maturation. Taken together, these findings suggest a critical role of E2-ERs system during oocyte meiotic progression and may propose a novel approach for oocyte *in vitro* maturation treatment in clinical practice.

In mammals, immature oocytes enter a specialized cell cycle (meiosis, which could reduce the number of chromosomes from diploid to haploid) during embryogenesis, but then pause at the diplotene stage of prophase around the time of birth for prolonged periods. In the case of women, oocytes may remain in this arrested state for 40 years or more. Until the puberty, the arrested oocytes in Graafian follicles resume meiosis in response to the pre-ovulatory luteinizing hormone (LH) surge stimulation, and then the mature oocytes (eggs) are ovulated into the oviduct to await fertilization. Oocytes arrested at the diplotene stage, which have an intact nuclear envelope are referred to as germinal vesicle (GV)-stage oocytes, and nuclear envelope dissolved (meiosis resumed) oocytes are referred to as GV breakdown (GVB)-stage oocytes.

It has been widely accepted that the key inhibitory substance for maintaining oocyte meiotic arrest in Graafian follicles is from follicular somatic cells ever since the experiments in the 1930s.^1^ Until recently, it is reported that this inhibitory signal in follicular mural granulosa cells (MGCs) is natriuretic peptide C (NPPC, also known as CNP), which could promote cyclic guanosine monophosphate (cGMP) production through binding to its receptor natriuretic peptide receptor 2 (NPR2) in cumulus cells (CCs).^2, 3, 4^ Cyclic GMP then diffuses into the oocyte via the large network of gap junction communications^5^ and inhibits phosphodiesterase 3A (PDE3A) activity, thereby suppressing the hydrolysis of cyclic adenosine monophosphate (cAMP)^6, 7^ and maintaining oocyte meiotic arrest by activating protein kinase A.^8, 9, 10^ Although the well understood downstream signaling, little is known about the events upstream of NPPC and NPR2 that maintain oocyte meiotic arrest. A recent study in the ovary suggests a potential role for LH in decreasing NPPC secretion and NPR2 guanylyl cyclase activity to promote meiotic resumption,^11, 12, 13, 14^ but molecular signaling that directly controls the expression of NPPC/NPR2 system is totally unknown.

Estradiol (E2), which is primarily produced by pre-ovulatory follicles under the influence of follicle-stimulating hormone (FSH),^15^ has a critical role in the development and maintenance of female reproductive organs. E2 exerts its effects by binding to its nuclear receptor proteins (estrogen receptors (ERs), including ER*α* and ER*β*, whose corresponding genes are named as *Esr1* and *Esr2*), which display cell-dependent and promoter context-dependent transcriptional activities. Adult mice lacking ER*α* (ER*α* knockout (*α*ERKO)) are infertile and possess ovaries that exhibit invariably elevated steroid synthesis and multiple hemorrhagic/cystic follicles.^16^ In contrast, adult mice lacking ER*β* (*β*ERKO) are subfertile and possess ovaries that show consistently reduced numbers of growing follicles and corpora lutea.^17^ Although dozens of evidences have shown that E2-ERs system has a vital role during folliculogenesis by enhancing the actions of FSH,^18, 19, 20, 21^ little is known about the functional role of E2-ERs system during the process of oocyte maturation.

Female fertility in mammals depends on the coordinated development of ovarian follicles and oocytes, which is regulated by two pituitary derived gonadotrophins (FSH and LH) during each reproductive cycle.^22^ It is widely accepted that LH is primarily responsible for the stimulation of meiotic resumption and the subsequent ovulation in pre-ovulatory follicles, and FSH stimulates the growth and development of the next wave of follicles.^23^ Based on this concept, FSH and LH are widely used in human and livestock to control ovarian superovulation and *in vitro* fertilization (IVF).^24, 25, 26^ Thus, understanding the mechanisms of FSH and LH controlling follicular development and oocyte maturation is critical for improving the effectiveness of assisted reproduction techniques (ARTs) in clinical applications. In addition, series of studies have indicated that FSH alone is sufficient to induce oocyte maturation *in vitro*,^27, 28, 29, 30^ which is contradictory with that *in vivo*. However, the related mechanisms are poorly understood.

In this study, our results revealed the novel role of E2-ERs in regulating oocyte meiotic resumption in mammals. We provided the experimental evidences showing that E2 and its nuclear receptors in granulosa cells, which are regulated by gonadotropins, govern oocyte meiotic progression by directly regulating *Nppc*/*Npr2* gene transcription in mouse and human ovaries. Elucidation of the physiological role of E2-ERs during the process of oocyte maturation will provide potential therapeutic targets in the treatment of oocyte *in vitro* maturation (IVM) in clinical applications.

## Results

### Gonadotropins control the expression of ERs *in vivo*

**Figure 1:**
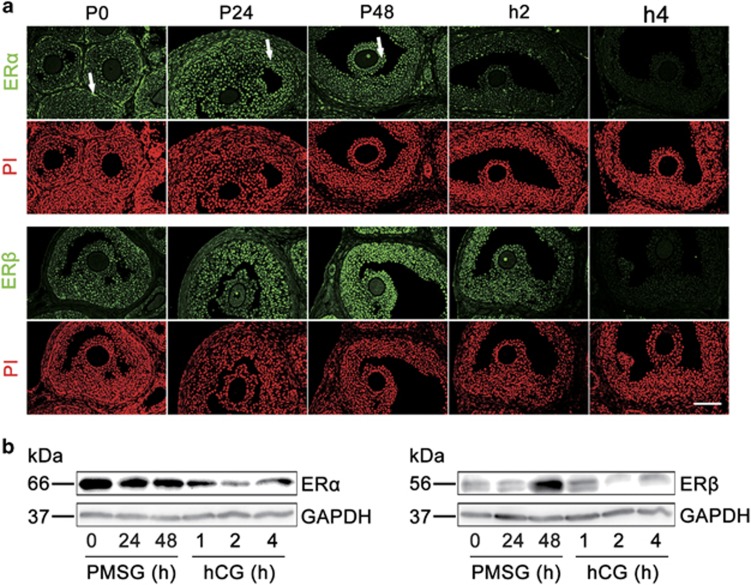
Gonadotropins control ER levels in mouse ovaries *in vivo*. (**a**) Immunofluorescence analysis of ER*α* and ER*β* expression in ovaries. Ovaries were stained for ER*α* or ER*β* (green) and the nuclear marker propidium iodide (PI, red) at the indicated time points after PMSG stimulation followed at 48 h later with hCG. ER*α* protein was highly expressed in theca cells (indicated by arrows in P0) in small follicles, but also stained in MGCs (indicated by arrows in P24) and CCs (indicated by arrows in P48) of large antral follicles by PMSG stimulation. ER*β* staining was predominantly observed in MGCs and CCs in large antral follicles. P, means PMSG, h means hCG. Scale bars: 100 *μ*m. (**b**) WB data indicating the regulation of gonadotropins on ER*α* and ER*β* levels in ovaries. Ovaries were isolated from 22- to 24-day-old mice stimulated with PMSG followed at 48 h later with hCG as indicated in figures. GAPDH served as a loading control

To explore the role of E2 during oocyte meiotic progression under physiological conditions, we first analyzed the localization of ER*α* and ER*β* in mouse ovaries. Immunofluorescence analysis (Figure 1a) of ovaries revealed that ER*α* was highly expressed in the theca cells (indicated by arrows in P0) of small follicles, but was also present in the MGCs (indicated by arrows in P24) and CCs (indicated by arrows in P48) of large antral follicles following follicular development by pregnant mare's serum gonadotropin (PMSG) stimulation. In contrast, ER*β* was predominantly observed in MGCs and CCs of large antral follicles. The localization of ER*α* and ER*β* in MGCs and CCs was substantiated by the immunohistochemistry analysis (Supplementary Figure S1).

We next analyzed the variation of ER*α* and ER*β* protein levels in mouse ovaries in respond to gonadotropin stimulation. As shown in Figure 1b, the whole ovarian content of ER*α* was expressed at a relatively high level after stimulation with PMSG, whereas ER*β* levels were significantly elevated after stimulation for 48 h by PMSG. Subsequently, the following human chorionic gonadotropin (hCG) significantly decreased the expression of ER*α* and ER*β* protein levels. The results of immunofluorescence analysis also confirmed the regulating patterns of gonadotropins on ER*α* and ER*β* levels (Figure 1a). On the other hand, the levels of NPPC and NPR2 in ovaries regulated by gonadotropins exhibited a similar expression pattern to that of ER*α* and ER*β* (Supplementary Figure S2), indicating a potential role of E2-ERs in maintaining oocyte meiotic arrest.

### E2-ERs promote and maintain *Nppc*/*Npr2* levels and oocyte meiotic arrest

**Figure 2:**
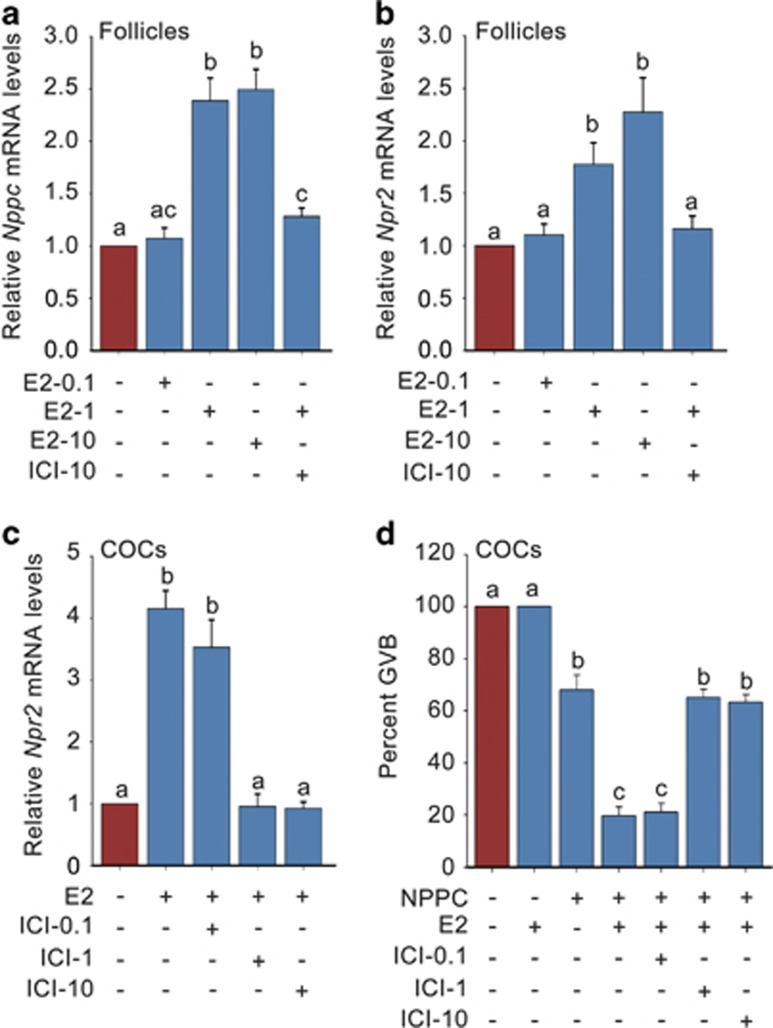
E2-ERs promote *Nppc*/*Npr2* levels and maintain NPPC-mediated oocyte meiotic arrest. (**a** and **b**) Effects of E2 and ICI182780 on *Nppc* and *Npr2* mRNA levels in follicles. Follicles were cultured in medium containing 0.0 *μ*M (control)–10.0 *μ*M E2 or plus 10 *μ*M ICI182780 (ICI: a nonselective ER*α* and ER*β* antagonist) for 4 h. *n*=3. (**c**) Effects of E2 and ICI on *Npr2* mRNA levels in COCs. COCs were cultured for 24 h in medium without (control) or with 0.1 *μ*M E2 or plus 0.1–1.0 *μ*M ICI. *n*=3. (**d**) Effects of E2 and ICI on NPPC-mediated oocyte meiotic arrest in COCs. COCs were cultured for 24 h and the percentages of oocytes that underwent GVB were determined. E2, 0.1 *μ*M; NPPC, 0.03 *μ*M; ICI, 0.1–1.0 *μ*M. *n*=4. Data represent the mean±S.E.M. Different letters (a-c) indicate significant differences between groups (*P*<0.05, ANOVA and Holm–Sidaik test)

To ascertain the role of E2-ERs system in controlling oocyte meiotic progression and the related mechanisms, we cultured follicles and cumulus–oocyte complexes (COCs) with E2 and ICI182780 (ICI: a nonselective ER*α* and ER*β* antagonist) *in vitro*. The results showed that E2 dose-dependently promoted *Nppc*/*Npr2* mRNA levels in follicles (Figures 2a and b) and significantly elevated *Npr2* mRNA levels in COCs (Figure 2c). The highest concentration of E2 (10.0 *μ*M) triggered close to 2.5-fold increase in *Nppc*/*Npr2* gene expression in follicles, and 0.1 *μ*M E2 induced a fourfold higher increase in *Npr2* levels in COCs. Importantly, the E2-elevated *Npr2* levels significantly promoted NPPC-maintained meiotic arrest in COCs after a culture of 24 h (Figure 2d). However, the E2 increased *Nppc*/*Npr2* levels and percentages of meiotic arrested oocytes were completely reversed by ICI either in follicles or in COCs (Figures 2a–d). Therefore, we conclude that E2-ERs act as an important role in maintaining oocyte meiotic arrest by mediating the signaling network between gonadotropins and NPPC/NPR2.

### ERKO mice exhibit increased percentages of meiotic resumed oocytes

**Figure 3:**
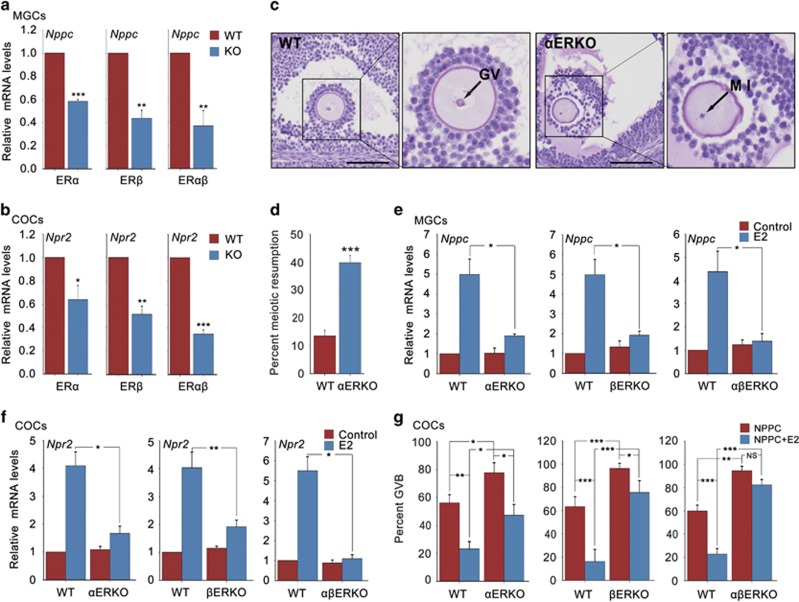
ERKO mouse ovaries show decreased NPPC/NPR2 levels and percentages of meiotic arrested oocytes. (**a** and **b**) Expression of *Nppc* levels in MGCs and *Npr2* levels in COCs isolated from 22- to 24-day-old WT and ERKO mouse ovaries. Ovaries were stimulated with PMSG for 46 to 48 h. *n*=3. (**c**) A prophase-arrested oocyte (GV) within a large antral follicle of a WT ovary and an oocyte with metaphase I (MI) chromosomes within a large antral follicle of a *α*ERKO ovary. Scale bars: 100 *μ*m. (**d**) Percentages of oocytes that had resumed meiosis, counted in serial sections of ovaries from WT and *α*ERKO mice. *n*=9. (**e** and **f**) Effects of E2 on *Nppc* levels in MGCs and *Npr2* levels in COCs isolated from WT and ERKO mice. MGCs and COCs were cultured for 24 h in medium without (control) or with 0.1 *μ*M E2. *n*=3. (**g**) Effect of E2 on NPPC-maintained oocyte meiotic arrest within COCs isolated from WT and ERKO mice. COCs were incubated in medium containing 0.03 *μ*M NPPC or plus 0.1 *μ*M E2 for 24 h. *n*=4. Data represent the mean±S.E.M. *P<0.05, **P<0.01 and ***P<0.001 (*t*-test)

If E2-ERs participate in the maintenance of oocyte meiotic arrest by promoting NPPC/NPR2 expression, then NPPC/NPR2 levels in ERKO mice should be decreased and oocytes within ERKO mice should exhibit a failure of meiotic arrest. To test our hypothesis and to study, which subtype of ERs is required in this process, we measured *Nppc*/*Npr2* mRNA levels in ERKO mice. As expected, the levels of *Nppc* in MGCs and *Npr2* in COCs from *α*ERKO, *β*ERKO and ER*α* and ER*β* double knockout (*αβ*ERKO) mouse ovaries were significantly decreased under physiological conditions, when comparing with that in wild-type (WT) mouse ovaries (Figures 3a and b). Thus, ER*α* and ER*β* are both essential for promoting NPPC and NPR2 levels.

We then analyzed the oocyte meiotic progression in ERKO mice. As expected, 39.8±2.6% of oocytes within the large antral follicles of *α*ERKO ovaries by PMSG stimulation had precociously resumed meiosis when comparing with 13.5±2.0% of WT oocytes (Figures 3c and d). However, as most of the follicles in *β*ERKO and *αβ*ERKO ovaries cannot develop to the pre-ovulatory stage (Supplementary Figure S4) resulting from the attenuated ovarian responsiveness to FSH/PMSG stimulation,^31, 32^ additional studies to test our hypothesis under physiological conditions are impossible to perform. Hence, we chose *in vitro* model as an alternative approach to investigate our hypothesis. As shown in Figures 3e and f, when MGCs and COCs were cultured *in vitro*, E2 induced approximately four to fivefold higher levels of *Nppc* and *Npr2* mRNA in WT mice, however, the E2-elevated *Nppc* levels in MGCs were significantly compromised in *α*ERKO mice (close to 1.9-fold) and not observed in *β*ERKO and *αβ*ERKO mice. Similarly, the *Npr2* mRNA levels promoted by E2 in COCs were remarkably compromised in *α*ERKO and *β*ERKO mice (close to 1.7-fold) and not observed in *αβ*ERKO mice. As MGCs has a relative lower NPR2 levels than CCs,^33^ we also examined *Npr2* mRNA levels in MGCs and found a similar *Npr2* expression pattern as that in COCs (Supplementary Figure S3). Moreover, when COCs were cultured *in vitro*, the majority of WT oocytes were maintained at meiotic arrested stage by E2 and NPPC and only 16.2±6.4% to 23.2±5.2% of oocytes resumed meiosis. In comparison, only few of the ERKO oocytes were maintained at meiotic arrested stage by E2 and NPPC, most of the oocytes had underwent GVB (*α*ERKO: 47.4±7.2% *β*ERKO: 75.6±9.3% *αβ*ERKO: 82.4±4.6%) (Figure 3g). Collectively, our genetic evidences show that both ER*α* and ER*β* are required for E2 to promote NPPC/NPR2 levels and maintain oocyte meiotic arrest under physiological conditions.

### ERs directly regulate *Nppc* and *Npr2* gene transcription

**Figure 4:**
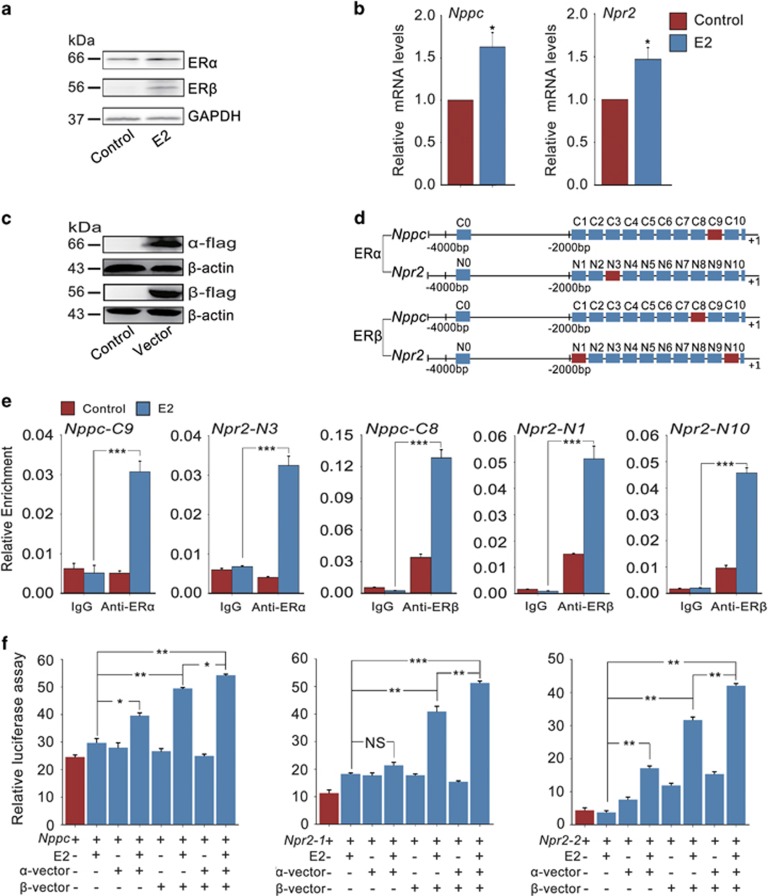
ERs directly regulate *Nppc* and *Npr2* gene transcription. (**a**) Expression of ER*α* and ER*β* proteins in KK1 cells in response to E2 stimulation. KK1 cells were treated without (control) or with 0.1 *μ*M E2 for 24 h. GAPDH served as a loading control. (**b**) Effect of E2 on *Nppc* and *Npr2* levels in KK1 cells. KK1 cells were cultured for 24 h in medium containing 0.1 *μ*M E2 or not (control). *n*=3. (**c**) Expression of ER*α* and ER*β* proteins in KK1 cells after transfection with empty vector (control), flag-tagged mouse *α*-vector or *β*-vector for 48 h. ER*α* and ER*β* protein levels were assessed using an anti-flag antibody. *β*-Actin served as a loading control. (**d**) Schematic diagram of *Nppc* and *Npr2* genome structure. Each rectangle denotes 200 bp. Red rectangles represent the *Nppc* or *Npr2* promoter binding sequences for ER*α* or ER*β*. (**e**) ChIP-qPCR analysis of the interaction between ER*α*/ER*β* proteins and *Nppc*/*Npr2* promoters in KK1 cells. KK1 cells (transfection with empty vector, flag-tagged mouse *α*-vector or *β*-vector) were treated without (control) or with 0.1 *μ*M E2 for 6 h. *n*=3. (**f**) The binding of ER*α*/ER*β* to *Nppc*/*Npr2* promoter regions detected by luciferase reporter assay. KK1 cells (transfection with pRL-TK plus flag-tagged mouse *α*-vector, *β*-vector or both) were treated without or with 0.1 *μ*M E2 for 24 h. pRL-TK, an internal control plasmid to normalize firefly luciferase activity of the reporter plasmids. *Nppc*, *Npp2*-*1* or *Npr2*-*2* represent pGL3-basic plasmid containing −2000 to 1 regions of the *Nppc* gene, −616 to 1 or −2000 to −1260 regions of the *Npr2* gene, respectively. *n*=4. Data represent the mean±S.E.M. *P<0.05, **P<0.01 and ***P<0.001 (*t*-test)

To uncover the potential mechanism how ERs regulate the expression of NPPC/NPR2 during oocyte meiotic progression, a mouse gonadotropin-responsive granulosa cell line (KK1)^34^ responding to E2 stimulation (Figures 4a and b) was used for chromatin immunoprecipitation (ChIP) analysis. Transfection of flag-tagged mouse ER*α*- and ER*β*-vector (*α*-vector and *β*-vector) was efficient in KK1 cells (Figure 4c). The DNA fragments that immunoprecipitated with the anti-flag antibody were PCR-amplified with 11 pairs of primers within the 4000-bp regions of *Nppc* and *Npr2* promoter sequences (Figure 4c). Results indicated that ER*α* binds to the −400 to −200 (C9) and −1600 to −1400 (N3) sequences of *Nppc* and *Npr2* promoters from the transcription start (+1), respectively. In contrast, ER*β* binds to the −600 to −400 (C8), −2000 to −1800 (N10) and −200 to 0 (N1) regions of the *Nppc* and *Npr2* promoters, respectively (Figures 4d and e).The putative binding sites of *Nppc* and *Npr2* promoter sequences for ER*α* and ER*β* analyzed *in silico* were shown in Supplementary Figure S5.

To further confirm the interaction between ER*α*/ER*β* proteins and *Nppc*/*Npr2* promoters, a dual-luciferase assay using reporter constructs containing the C8, C9, N1, N3 and N10 loci of *Nppc* or *Npr2* promoter was performed (Figure 4f). Transfection of mouse *α*-vector, *β*-vector, in particular, co-transfection of both *α*-vector and *β*-vector significantly enhanced luciferase activity in response to E2 treatment, demonstrating that ER*α* and ER*β* collaboratively regulate NPPC/NPR2 levels through binding to the promoter regions of *Nppc* and *Npr2* genes directly.

### E2-ERs promote *Nppc* and *Npr2* levels in human granulosa cells

**Figure 5:**
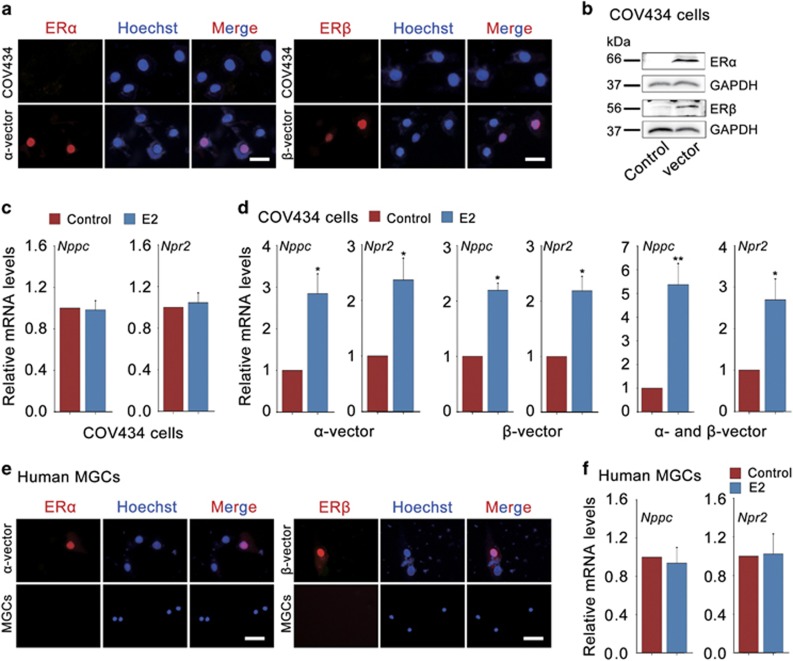
E2-ERs promote *Nppc* and *Npr2* levels in human granulosa cells. (**a**) Expression of ER*α* and ER*β* (red) in COV434 cells (upper) or COV434 cells after transfection with myc-tagged human *α*-vector or *β*-vector for 48 h (under). The nuclei were stained as blue by Hoechst. Scale bars: 25 *μ*m. (**b**) WB analysis of ER*α* and ER*β* protein levels in COV434 cells. COV434 cells were transfected with empty vector (control), myc-tagged human *α*-vector or *β*-vector for 48 h. GAPDH served as a loading control. (**c**) E2 failed to promote *Nppc* and *Npr2* mRNA levels in COV434 cells. COV434 cells were cultured in medium without (control) or with 0.1 *μ*M E2 for 24 h. Data represent the mean±S.E.M. *n*=3. (**d**) E2 increased *Nppc* and *Npr2* mRNA levels in COV434 cells after transfection with myc-tagged human *α*-vector, *β*-vector or both. COV434 cells were incubated without (control) or with 0.1 *μ*M E2 for 24 h. *P<0.05 and **P<0.01 (*t*-test). Data represent the mean±S.E.M. *n*=3. (**e**) Expression of ER*α* and ER*β* (red) in human MGCs. Human MGCs were freshly isolated from ovulatory follicles, which were stimulated with FSH for 10 days, and followed by LH for 36 h. Human MGCs after transfection with myc-tagged human *α*-vector or *β*-vector for 48 h served as the corresponding positive control (upper). The nuclei were stained as blue by Hoechst. Scale bars: 25 *μ*m. (**f**) Effect of E2 on *Nppc* and *Npr2* mRNA levels in human MGCs. Human MGCs were freshly isolated from ovaries stimulated with FSH for 10 days followed by LH for 36 h and cultured for 24 h in medium containing 0.1 *μ*M E2 or not (control). Data represent the mean±S.E.M. *n*=3

To investigate whether E2-ERs are involved in the progress of human oocyte meiosis, we used a human granulosa cell line (COV434), which keeps most of the biological characteristics of human granulosa cells.^35^ Results indicated that as the deficiency of ER proteins (Figures 5a and b), E2 failed to promote *Nppc*/*Npr2* levels in COV434 cells (Figure 5c). Interestingly, when ER*α* and ER*β* levels were over expressed in COV434 cells by transfecting with myc-tagged human *α*-vector or *β*-vector (Figures 5a and b), E2 significantly promoted *Nppc*/*Npr2* levels (Figure 5d), COV434 cells transfected with empty vector served as a control (Supplementary Figure S6). Therefore, we conclude that E2-ERs also have a critical role in regulating *Nppc*/*Npr2* levels in human granulosa cells.

To study the function of E2-ERs during human oocyte meiotic progression under more physiological conditions, we measured *Nppc*/*Npr2* levels regulated by E2-ERs in human MGCs, which were freshly isolated from ovulatory follicles. As previous reported that human granulosa cells have the most intense staining of ERs in pre-ovulatory follicles before LH surge (when FSH expression is highest) but not detectable ER proteins after LH surge during the menstrual cycle.^36^ Here we also found no detectable ER proteins in human MGCs from ovulatory follicles, which were simulated with FSH for 10 days and followed by LH for 36 h, thereby E2 failed to maintain *Nppc*/*Npr2* levels (Figures 5e and f), which are similar to the results in mouse (Figures 1,2 and 3). As a result of the limitation of ethics, here we were unable to obtain human MGCs (which have ER staining) stimulated by FSH only. Alternatively, human MGCs after transfection with myc-tagged human *α*-vector or *β*-vector for 48 h were used as the corresponding positive control (Figure 5e). Taken together, gonadotropins also control ER levels in human MGCs, which can promote NPPC/NPR2 levels in response to E2 treatment, indicating a potential role for E2-ERs in governing the process of human oocyte maturation.

### Gonadotropins induce oocyte maturation by suppressing ER levels

**Figure 6:**
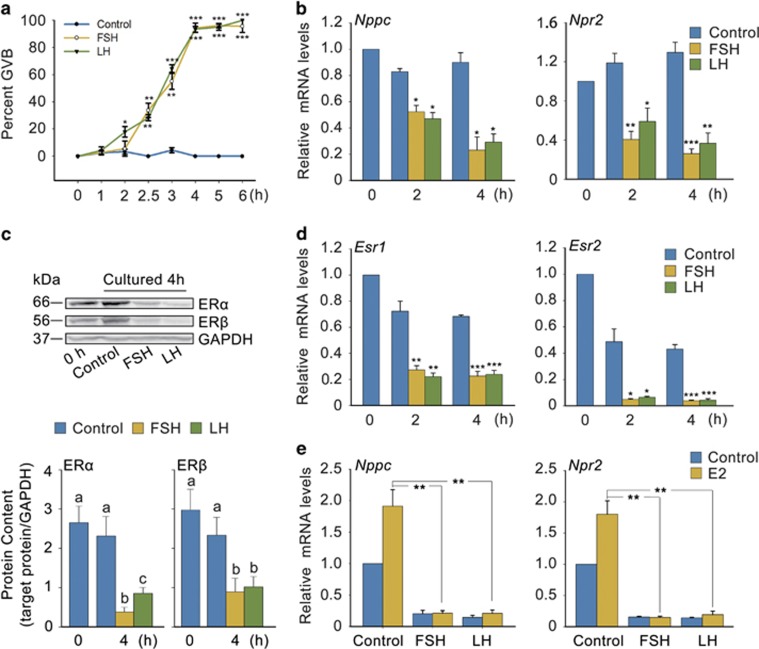
FSH and LH induce oocyte maturation by downregulating ER levels *in vitro*. (**a**) Effects of FSH and LH on oocyte maturation (referred to as GVB) in follicles. Freshly isolated follicles (0 h) were cultured in medium containing FSH or LH for the indicated period of time. *P<0.05, **P<0.01 and ***P<0.001 *versus* corresponding control (*t*-test). *n*=6. (**b**) Effects of FSH and LH on *Nppc* and *Npr2* mRNA levels in follicles cultured for the indicated period of time. *P<0.05, **P<0.01 and ***P<0.001 *versus* corresponding control (*t*-test). *n*=3. (**c**) FSH and LH decreased ER*α* and ER*β* levels in follicles. GAPDH served as a loading control. Graph shows the density quantification of WB band (normalization of target protein with respect to GAPDH). Bars represent mean of relative abundance, different letters (a-c) indicate significant differences between groups (*P*<0.05, ANOVA and Holm–Sidak test). (**d**) FSH and LH decreased *Esr1* and *Esr2* mRNA levels in follicles. *Esr1* and *Esr2* are the corresponding gene names of ER*α* and ER*β*. *P<0.05, **P<0.01, ***P<0.001 *versus* corresponding control (*t*-test). *n*=3. (**e**) E2 failed to promote *Nppc* and *Npr2* mRNA levels in follicles primed with FSH or LH for 4 h. **P<0.01 (*t*-test). *n*=3. Data represent the mean±S.E.M. FSH, 0.1 IU/ml; LH, 1.0 *μ*g/ml; E2, 1.0 *μ*M

To investigate whether E2-ERs are responsible for gonadotropin-induced oocyte maturation, we cultured follicles with FSH and LH *in vitro*. Both FSH and LH time-dependently promoted oocyte meiotic resumption in follicles, and >90% GVB were completed after only 4 h (Figure 6a; Supplementary Figure S7A). Furthermore, *Nppc*/*Npr2* mRNA levels and ER levels in follicles were time-dependently reduced along with the progress of oocyte meiotic resumption in respond to FSH and LH stimulation, even when E2 was added (Figures 6b–e; Supplementary Figure S7B), which was similar to that LH does *in vivo* (Figure 1b; Supplementary Figure S2). These results indicated that gonadotropins induce oocyte maturation may by suppressing ER levels, which directly control NPPC and NPR2 levels.

To further confirm our hypothesis, we cultured COCs with FSH *in vitro*. As shown in Supplementary Figure S7C, FSH significantly promoted oocyte meiotic resumption, which were suppressed by NPPC alone or together with E2. Further results indicated that FSH markedly decreased the expression of *Esr*1 and *Esr*2 mRNA levels in COCs (Supplementary Figure S7D), in turn decreasing *Nppc* and *Npr2* levels, even when E2 was added (Supplementary Figure S7E). Effect of LH was not assessed because the deficiency of LH receptor in COCs. Overall, these data suggested that the decrease of ER levels is required for gonadotropins to promote oocyte meiotic resumption.

In view of our previous results (Figure 1; Supplementary Figure S2), here we found that the effect of LH on ER levels, in turn on NPPC/NPR2 levels and oocyte maturation *in vitro* was similar to that *in vivo*. Interestingly, expression of ER and NPPC/NPR2 levels and oocyte maturation regulated by FSH *in vitro* were opposite from that *in vivo* (Figure 6; Supplementary Figure S7). Therefore, the different effects of FSH on oocyte maturation *in vivo versus in vitro* are likely result from its opposite regulating patterns on ER levels, reinforcing the physiological significance of E2-ERs governing oocyte meiotic resumption.

## Discussion

**Figure 7:**
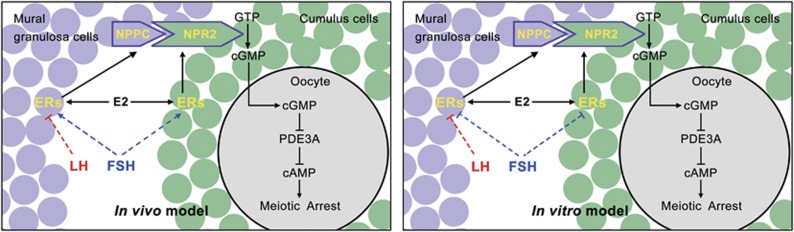
Model depicting the role of E2-ERs in governing oocyte meiotic resumption in response to gonadotropin stimulation. FSH stimulates ER levels in granulosa cells *in vivo*, which can directly promote *Nppc* and *Npr2* gene transcription in response to E2 stimulation, thus raising cGMP levels in CCs. Cyclic GMP thereby diffuses into the oocyte through gap junction and inhibits PDE3A activity and cAMP hydrolysis and maintains oocyte meiotic arrest. Conversely, LH decreases ER*α* and ER*β* levels both *in vivo* and *in vitro* as FSH does *in vitro*, in turn decreasing NPPC/NPR2 and cGMP levels, thus triggering the hydrolysis of cAMP by PDE3A and oocyte resumes meiosis

It has been known for many years that successful oocyte maturation relies on close communication and cooperation between the oocyte and surrounding granulosa cells, which is regulated by various endocrine, paracrine and autocrine factors. Here we show that the well-known female hormone (E2) and its nuclear receptors in granulosa cells, which are controlled by gonadotropins, have an important role in governing oocyte meiotic resumption in mouse and human ovaries. Figure 7 demonstrates a working model of this process based on our findings presented here and previous studies. Briefly, FSH stimulates the production and expression of E2 and ERs *in vivo*, E2-ERs then upregulate NPPC and NPR2 levels by directly binding to *Nppc* and *Npr2* promoter regions. The elevated NPPC and NPR2 thus maintain oocyte meiotic arrest by stimulating cGMP production.^2, 6, 7^ Conversely, pre-ovulatory LH surge decreases ER levels *in vivo* as FSH and LH do *in vitro*, thus decreasing NPPC and NPR2 levels, thereby triggers oocyte meiotic resumption. Therefore, E2-ERs act as a vital role during the process of oocyte maturation.

In mammals, oocyte meiotic arrest is important for sustaining the oocyte pool,^37^ which determines the entire reproductive potential of female over the life span. Recent studies indicate that this blockade in oocyte meiotic cell cycle is under the control of NPPC/NPR2 system in follicular somatic cells.^2, 3^ In addition, NPPC/NPR2 have a variety of biological roles involved in reproduction, such as fetal development, pre-eclampsia and intrauterine growth retardation during pregnancy.^38^ This system has also been shown to modulate spermatozoa motility, testicular germ cell development and testosterone synthesis in male testis.^39, 40^ However, less is known about how signaling upstream of NPPC/NPR2 system regulates these processes. In this study, we provide direct evidence indicating that E2-ERs promote *Nppc* and *Npr2* gene transcription by directly binding to their promoter regions. Moreover, mice deficient in either ER*α* or ER*β* showed a substantial decrease in *Nppc*/*Npr2* mRNA levels *in vivo*, and E2-promoted *Nppc*/*Npr2* mRNA levels in WT mice were significantly compromised in ERKO mice *in vitro*. Hence, we conclude that E2-ERs are vital upstream regulators of NPPC/NPR2.

Given the indispensable role of NPPC/NPR2 in maintaining oocyte meiotic arrest,^2, 3^ regulation of NPPC/NPR2 by E2-ERs would be futile under physiological conditions if meiotic progression were not affected in ERKO mice. Indeed, oocytes within Graafian follicles of *α*ERKO mice exhibited precocious gonadotropin-independent meiotic resumption. However, additional studies to test our hypothesis under physiological conditions are difficult to perform because ovaries of ERKO mice exist in a severely abnormal hormonal environment and show impaired development.^41^ This is particularly true for *β*ERKO mice because oocyte growth, follicular development and ovarian response to gonadotropins are seriously attenuated in these mice.^31, 32^ Consistent with these studies, we find that the majority of follicles from *β*ERKO and *αβ*ERKO mice stimulated by PMSG failed to develop to the pre-ovulatory stage, as manifested by reduced ovarian volume, weight and numbers of large antral follicles and isolated COCs (Supplementary Figure S4). As an alternative approach, COCs were used to explore the function of ERs during oocyte maturation, results indicate that meiotic arrest maintained by NPPC alone or together with E2 in WT COCs were remarkably compromised in *α*ERKO, especially in *β*ERKO and *αβ*ERKO COCs *in vitro*. Therefore, we conclude that ER*β* is critical for FSH-stimulated oocyte growth and follicular development, and both ER*α* and ER*β* are essential for maintaining NPPC/NPR2-mediated oocyte meiotic arrest.

Ovulation of a fertilizable egg involves in successful follicular development and oocyte meiotic resumption, which are regulated by FSH and LH.^22, 42^ Inappropriate follicular development and oocyte maturation may cause reproductive disorders, such as polycystic ovarian syndrome or premature ovarian failure. Here we show that FSH promotes expression of ER levels in follicular granulosa cells accompanying with E2 synthesis,^31, 43^ E2-ERs then promote NPPC/NPR2 levels by binding to *Nppc* and *Npr2* promoter regions and maintain oocyte meiotic arrest. On the other hand, E2 enhances FSH-induced granulosa cell proliferation, maturation of the whole follicle to a pre-ovulatory state and the LH signaling pathway to expel a healthy oocyte.^31, 44^ In addition, the elevated NPPC/NPR2 by E2-ERs can stimulate pre-antral and early antral follicles develop to the pre-ovulatory stage.^45^ By contrast, LH significantly decreases ER levels, thus decreasing NPPC/NPR2 levels and inducing oocyte maturation. Interestingly, FSH also induces meiotic resumption by suppressing ER levels *in vitro*. Therefore, we conclude that E2-ERs are key regulators mediating gonadotropin-controlled follicular development and oocyte meiotic resumption.

This study answers a controversial issue about the different effects of FSH on oocyte maturation *in vivo versus in vitro*. It is reported that FSH is primarily responsible for the development of pre-antral follicles and selection of dominant follicles under physiological conditions.^23^ However, series of studies have indicated that FSH alone is sufficient to induce oocyte maturation *in vitro*,^27, 28, 29, 30^ which is contradictory to *in vivo* situation. Our results that FSH could induce meiotic resumption in follicles and COCs also confirm the functional role of FSH in promoting oocyte maturation (Figure 6; Supplementary Figure S7). We then provide direct evidences that FSH promotes oocyte maturation *in vitro* by decreasing ER levels as LH does, implying that LH has no bias, whereas FSH has opposite regulating patterns toward to ER levels *in vivo versus in vitro*. Therefore, we hypothesize that the different effects of FSH on oocyte maturation *in vivo versus in vitro* may result from its opposite regulating patterns on ER levels, confirming the significant role of E2-ERs during the process of oocyte maturation. However, additional studies are required to explore the exact molecular mechanisms by which ER proteins are regulated.

In human, immature oocytes in pre-ovulatory follicles can experience IVM and IVF, thus initiating pregnancy.^46, 47^ As a result of the advantages of having a lower risk of ovarian hyperstimulation syndrome,^46^ the IVM procedure is more and more favored by infertility patients. Here our results indicate that gonadotropins induce oocyte maturation by decreasing ERs-controlled NPPC/NPR2 levels in mice. Moreover, we find that E2-ERs also control NPPC/NPR2 expression and LH decreases ER levels in human MGCs. Considering previous reports that pre-ovulatory LH/hCG surge decreases NPPC secretion in human follicular fluid,^11^ we hypothesize that the E2-ERs system may also control the resumption of human oocyte meiosis in response to gonadotropin stimulation by regulating NPPC and NPR2 levels. This may provide a novel approach for oocyte IVM treatment in clinical practice.

In conclusion, this study identifies a potential role of E2-ERs in controlling oocyte meiotic resumption in mouse and human ovaries by linking the signaling networks between gonadotropins and NPPC/NPR2. These findings may not only contribute to a more comprehensive understanding of mammalian oocyte and follicular development but also propose a novel approach for the treatment of oocyte IVM in clinical practice.

## Materials and methods

### Mice

C57BL/6 female mice obtained from the Laboratory Animal Center of the Institute of Genetics and Developmental Biology (Beijing, China) were used in animal experiments. *α*ERKO^16^ and *β*ERKO^17^ mice were obtained from The Jackson Laboratory (Bar Harbor, ME, USA), as previously described. All animal procedures were approved by the Use Committee of China Agricultural University and performed in accordance with the guidelines and regulatory standards of the Institutional Animal Care and Use of Animals for Scientific Purposes.

### Human

Human MGCs were obtained from 10 patients, aged 25–40 years, who underwent conventional IVF-embryo transfer or intracytoplasmic sperm injection (ICSI) treatments at the Peking University First Hospital, Medical Center of Reproduction and Genetics (Beijing, China). The study was approved by the Peking University First Hospital committee on Human Research, and informed consent was obtained from all patients involved in this study. The purified human MGCs were cultured in M199 medium containing 10% fetal calf serum as previously described.^14^

### Chemicals, hormones and media

Unless otherwise noted, all chemicals were purchased from Sigma-Aldrich (St. Louis, MO, USA). PMSG and hCG were obtained from Sansheng Pharmaceutical Co. Ltd (Ningbo, China), the concentration of both PMSG and hCG used for each mouse was 5 IU. Medium used for all experiments were obtained from Gibco (Life Technologies, Waltham, CA, USA).

### Immunohistochemistry and immunofluorescence

Ovaries and follicles were fixed in cold 4% paraformaldehyde, for 48 and 24 h, respectively, dehydrated in ethanol and toluene, embedded in paraffin and sectioned at 5 *μ*m onto APES-treated microscope slides (ZLI-9001, Zhongshan Company, Beijing, China) for immunohistochemistry^48^ or immunofluorescence staining,^49^ as previously described. Cells were fixed in cold 4% paraformaldehyde for 20 min, membrane osmosed in 0.1% triton X-100 for 5 min, blocked with 10% normal donkey serum for 1 h. COV434 cells and human MGCs transfected with vectors were treated at adherent state and freshly isolated human MGCs were treated at suspension state. Primary antibodies used for immunohistochemistry were diluted as follows: ER*α* (sc-542, 1:500; Santa Cruz Biotechnology, Santa Cruz, CA, USA) and ER*β* (sc-8974, 1:2000; Santa Cruz Biotechnology). Primary antibodies used for immunofluorescence were diluted as follows: ER*α* (sc-542, 1:100) and ER*β* (ab3576, 1:200; Abcam, Cambridge, UK). An isotype-matched IgG was used as the negative control.

### Western blotting (WB)

Total proteins were extracted in tissue and cell lysis solution for WB and immunoprecipitation (CellChip, BJ Biotechnology Co., Ltd, Beijing, China) and protein concentration was measured by the BCA protein assay kit as recommended by the manufacturer. WB was performed as previously described.^48^ GAPDH or *β*-actin served as a loading control. Primary antibodies used for WB were diluted as follows: ER*α* (sc-542, 1:200), ER*β* (sc-53494, 1:200; Santa Cruz Biotechnology) and DYKDDDDK (Flag) (AE005, 1:3000; ABclonal, Boston, MA, USA).

### qRT-PCR

Total RNA was extracted and reverse transcribed to cDNA, and qRT-PCR was performed to analyze gene expression changes as previous description.^49^ Expression data were normalized to the amount of *Gapdh* (mus) or *β-actin* (homo). Each experiment was repeated independently at least three times. Primer sequences used in qRT-PCR are listed in Supplementary Table S1.

### Isolation and culture of mouse MGCs, COCs and follicles

MGCs and COCs were collected from ovaries of 22- to 24-day-old mice stimulated with 5 IU PMSG for 46–48 h in M199 medium, which also contained 4 mM hypoxanthine to prevent oocyte maturation until they were distributed to experimental groups. Follicles (450–550 *μ*m in size) were isolated in Leibovitz's L-15 medium. Before distributing to the experimental groups, all samples were washed three times with the corresponding culture medium. COCs were cultured for 24 h in M199 medium, supplemented with 0.1 IU/ml FSH, 0.1 *μ*M E2, 0.03 *μ*M NPPC or 0.1–10.0 *μ*M ICI (S1191, Selleck Chemicals, Houston, TX, USA) as described in the Results section. MGCs were cultured using a monolayer culture system as described in previous study.^50^ Follicles were cultured for 0–6 h on an insert (PICMORG50, Millipore, Billerica, MA, USA) in 35 mm Petri dish with DMEM medium plus 0.1 IU/ml FSH, 1.0 *μ*g/ml LH, 0.1–1.0 *μ*M E2 or 10.0 *μ*M ICI as indicated in the Results section. At culture termination, oocyte maturation was assessed by scoring the released oocytes for GV or GVB after removal of CCs. All samples were immediately frozen in liquid nitrogen and stored at −80 °C until analysis for mRNA or protein analysis. Each experiment was repeated at least three times.

### Oocyte quantification

Ovaries from 22- to 24-day-old ERKO mice stimulated with 5 IU PMSG for 46–48 h were fixed, dehydrated, embedded in paraffin and serially sectioned at 5 *μ*m. Meiotic status within Graafian follicles was scored by examining serial sections through the entire ovary and stained with periodic acid/Schiff reagents and Lillie–Mayer hematoxylin. Healthy oocytes in every section were counted and the nucleus was scored only once per oocyte. If an intact nucleus (GV) was observed, it was scored as meiotic arrest. If the GV was no longer present or condensed chromosomes were visible in the oocytes, they were scored as having resumed meiosis. The criteria for classification of follicles were applied as previous reported.^51^

### Plasmids

The cDNA-derived mouse ER*α*/ER*β* and human ER*α*/ER*β* sequences were flag-tagged (mouse) and myc-tagged (human) at the N terminus and generated by PCR. Amplified fragments were cloned into pHAGE-CMV-MCS-PGK puro 3+3tag–anp32a plasmid (mouse, Promega, Madison, WI, USA) and phage-6Tag-puro-CMV plasmid (human, Promega). For the luciferase assay, the upstream regions of the *Npr2* gene relative to the transcription start site (+1) were divided into two fragments (*Npr2*-1: −616 to 1; *Npr2*-2: −2000 to −1260). The upstream regions (−2000 to 1) of the *Nppc* gene and each fragment of the *Npr2* gene were then amplified by PCR using mouse genomic DNA as a template. The amplified fragments were cloned into pGL3-basic plasmid (Promega). PCR primer sequences are listed in Supplementary Table S2.

### Cell culture, transfection and dual-luciferase reporter assay

KK1 cells were grown in DMEM medium containing 10% fetal calf serum and 300 mg/l G418 in an atmosphere of 5% CO_2_ and 95% air at 37 °C. COV434 cells were grown in McCoy's 5A medium with 10% fetal calf serum. Cells at a confluence of about 80% on plates were transfected with plasmid DNA using lipofectamine 3000 (Life Technologies) according to the manufacturer's instructions. Twenty-four hours after transfection, the medium was replaced with fresh medium or supplemented with alcohol (vehicle) or 0.1 *μ*M E2 and cultured for additional 6 h or 24 h as described in the Figure legends. Cells were collected for RNA or protein analysis.

For the luciferase assay, KK1 cells (transfection with mouse *α*-vector, *β*-vector or both) were maintained in medium supplemented with alcohol (vehicle) or 0.1 *μ*M E2. pRL-TK, an internal control plasmid expressing Renilla (Promega), was co-transfected into cells to normalize firefly luciferase activity of the reporter plasmids. Cells were collected after a culture of 24 h. Luciferase activities were measured using the Dual-Luciferase Reporter Assay System (Promega). Assays were performed at least three times, with each sample in duplicate.

### Chromatin immunoprecipitation

For the ChIP analysis, KK1 cells (transfection with mouse *α*-vector, *β*-vector or both) maintained in medium supplementing with alcohol (vehicle) or 0.1 *μ*M E2 were collected at 6 h, and then washed three times with ice-cold PBS. Cell pellets were lysed in 1% SDS buffer containing a protease inhibitor. Chromatin was sheared by sonication until the average DNA length was about 300–500 bp, as evaluated by 2% agarose gel electrophoresis. Sheared chromatin was diluted in ChIP dilution buffer to a final SDS concentration of 0.1%. Salmon sperm DNA/protein agarose slurry was added to preclear the chromatin solution. One percent of the chromatin fragments were stored at −20 °C to be used later for non-precipitated total chromatin (input) for normalization. Ninety-nine percent of the chromatin fragments were incubated with 3 *μ*g anti-flag antibodies overnight at 4 °C. Normal mouse IgG (Santa Cruz Biotechnology) was used as a negative control for nonspecific immunoprecipitation. The chromatin–antibody complex was incubated with protein A/G beads for 1 h at 4 °C. The antibody/DNA complex on the agarose beads was collected by centrifugation. Beads were washed with various buffers in the following order: low salt immune complex buffer, high salt immune complex buffer, LiCl immune complex buffer and TE buffer. Beads were suspended in elution buffer, and precipitated protein/DNA complexes were eluted from the antibodies/beads. The resulting protein/DNA complexes were subjected to cross-link reversal in 5 M NaCl at 65 °C for 10 h followed by addition of 0.5 M EDTA, 1 M Tris-HCl and 10 *μ*g/ml proteinase K at 55 °C for 2 h. DNA was purified by phenol/chloroform extraction and ethanol precipitation. qRT-PCR was used to detect immunoprecipitated chromatin fragments, as well as input chromatin using primers indicated in Supplementary Table S3.

### Statistical analysis

All experiments were performed at least three times, and results were expressed as the mean±S.E.M. Differences between two groups were analyzed by *t*-test using SigmaPlot software (Systat Software, Inc., San Jose, CA, USA). For experiment with more than two groups, differences between groups were analyzed by analysis of variance (ANOVA). When a significant F ratio was detected by ANOVA, the groups were compared using the Holm–Šidák test. Statistically significant values of *P*<0.05, *P*<0.01 and *P*<0.001 are indicated by one, two and three asterisks in the *t*-test, respectively. Different letters indicate significant differences between groups (*P*<0.05) in the ANOVA and Holm–Š idák test.
