# Supplementary material for: Estrogen receptors in granulosa cells govern meiotic resumption of pre-ovulatory oocytes in mammals
Source: Cell Death Dis. 2017 Mar 9;8(3):e2662–. doi: 10.1038/cddis.2017.82 (PMC5386574; doi:10.1038/cddis.2017.82)
Supplement: Supplementary Information [file cddis201782x1.pdf]

Supplemental Material

Wei Liu, et al.

Estrogen receptors in granulosa cells govern meiotic resumption of pre-ovulatory oocytes in mammals

Supplementary Information

Supplementary Figure Legends

Figures S1-S7

Supplementary Tables

Tables S1-S3

Supplementary References

Supplementary Information

Estrous cycle monitoring. Vaginal cells from 6 to 8 weeks female mice were collected by daily saline wash and analyzed for identification of estrous cycle stage as previously described.[1] Only those females displaying at least two successive 4- or 5-day vaginal cycles were used for the experiments.

Collection of plasma and follicular fluid. Blood samples from adult female mice at different estrous cycles were collected into 1.5 ml centrifuge tubes containing 7.5% EDTA and 0.6 TIU/ml aprotinin. After collection, tubes were gently rocked several times for anti-coagulation immediately, and then centrifuged at 1 600 g for 15 min at 4 ℃. Follicular fluid samples were collected into 35 mm Petri dishes containing 0.4 ml normal saline and 0.6 TIU/ml aprotinin by puncturing the follicles from adult mice at different estrous cycles or prepubertal mice stimulated with 5 IU pregnant mare's serum gonadotropin (PMSG) followed at 48 h later with 5 IU human chorionic gonadotropin (hCG). Liquid and ovarian fragments were then transferred into 1.5 ml centrifuge tubes and centrifuged at 3 000 rpm for 15 min at 4 ℃. After centrifugation, plasma and follicular fluid in the supernatant were collected and stored at -80 ℃ for radioimmunoassay (RIA) analysis.

RIA. The amount of NPPC protein present in plasma and follicular fluid was measured by RIA kit (RK-012-03, PHOENIX BIOTECH, Hubei, China). Synthetic CNP-22 (0-1280 pg/ml) was used as a standard. A standard curve was constructed using a log-linear curve fit with B/B0 (%) (y-axis, where B is the average cpm of the paired standards and B0 is the cpm of total activity) against NPPC concentration (x-axis). Values were normalized to the amount of protein (pg) present in the samples.

Supplementary Figure Legends

Figure S1. Expression of ERα and ERβ in pre-ovulatory follicles. Ovaries were harvested from 22- to 24-day-old mice stimulated by PMSG for 48 h and analyzed by immunohistochemistry staining with antibodies to ERα or ERβ. ERα protein was expressed in theca cells (indicated by arrows), mural granulosa cells (MGCs, indicated by *) and cumulus cells (CCs, indicated by triangle) of pre-ovulatory follicles. ERβ staining was predominantly observed in MGCs and CCs of pre-ovulatory follicles. An isotype-matched IgG was used as the negative control. The criteria for classification of follicles were applied as previous reported.[2] Scale bars: 100 μm.

Figure S2. Gonadotropins regulate NPPC and NPR2 levels *in vivo*. (A and B) *Nppc* and *Npr2* mRNA levels in 22- to 24-day-old mouse ovaries, which were stimulated with PMSG for 0, 24 or 48 h, and at 48 h, some having been injected with hCG for 2 or 4. n=3. (C) RIA analysis of NPPC protein levels (pg/ml) in follicular fluid from 22- to 24-day-old mice, which were stimulated with PMSG and followed at 48 h later with hCG. n=7. (D) NPPC protein levels (pg/ml) in mouse follicular fluid from adult mice at different estrous cycles. NPPC protein levels exhibited estrous cycle-dependent variations with maximal expression at proestrus (when FSH production is highest) compared with the relative sustained levels in plasma. P, proestrus; E, estrus; M, metestrus; D, diestrus. **, $p < 0.01$ vs. follicular fluid group in proestrus group (t-test). n=6.

Data represent the mean ± SEM. Different letters (a-e) indicate significant differences between groups ($p < 0.05$, ANOVA and Holm–Šidák test) in profiles in (A-C).

Figure S3. Effects of E2 on *Npr2* mRNA levels in MGCs isolated from WT and ERKO mice by RT-qPCR. MGCs, which were isolated from 22- to 24-day-old mice following PMSG stimulation for 46 to 48 h, were cultured in medium without (control) or with 0.1 μM E2 for 24 h *in vitro*. E2-elevated *Npr2* mRNA levels in WT mice were severely compromised in ERKO mice. Data represent the mean ± SEM, **, $p < 0.01$ (t-test). n = 4.

Figure S4. βERKO and αβERKO mice show an attenuated ovarian responsiveness to FSH/PMSG. Ovarian volume, weight and numbers of large antral follicles and isolated COCs in each ovary from 22- to 24-day-old βERKO (A) and αβERKO (B) mice treated with PMSG for 46 to 48 h were significantly decreased. Ovarian volume was approximated by the equation V= long diameter × short diameter^2.[3] Average ovarian diameter was determined under a graduated stereomicroscope. Ovarian weight was

manifested as wet weight. Data represent the mean ± SEM. *, $p < 0.05$ and ***, $p < 0.001$ (t-test). n=12. Scale bars: 2 mm.

Figure S5. Putative binding sites of *Nppc* and *Npr2* promoter sequences for ERα and ERβ. Putative binding sites were analyzed using the TRANS-FACV® gene tool software (http://www.gene-regulation.com).[4] Each rectangle denotes 200 bp. Red rectangles are *Nppc* or *Npr2* promoter binding sequences for ERα or ERβ. (A) The putative binding sites of *Nppc* and *Npr2* promoter sequences for ERα. (B) The putative binding sites of *Nppc* and *Npr2* promoter sequences for ERβ.

Figure S6. Expression of ERα/ERβ and *Nppc*/*Npr2* mRNA levels in COV434 cell line. (A) Immunofluorescence analysis of ERα and ERβ (red) expression in COV434 cells transfected with empty vector for 48 h. The nuclei were stained as blue by hoechst. Scale bars: 25 μm. (B) Expression of *Nppc* and *Npr2* mRNA levels in COV434 cells transfected with empty vector. Cells were cultured in medium without (control) or with 0.1 μM E2 for 24 h. Data represent the mean ± SEM. n=3.

Figure S7. FSH promotes oocyte meiotic resumption by decreasing ER levels *in vitro*. (A) FSH promoted oocyte meiotic resumption (referred to as GVB) in follicles. Follicles were cultured for 4 h in medium containing 0.0-0.1 IU/ml FSH. At least 30 follicles were assessed in each group. **, $p < 0.01$ vs. Con.. n=3. (B) FSH and LH decreased ERα and ERβ (green) protein levels in follicles after a culture of 4 h. The nuclei were stained as red by propidium iodide (PI). Scale bars: 100 μm. (C) FSH induced oocyte maturation in COCs, which was suppressed by NPPC alone or plus E2. COCs were cultured for 24 h. n=4. (D) FSH decreased *Esr1* and *Esr2* mRNA levels in COCs after a culture of 24 h. *Esr1* and *Esr2* are the corresponding gene names of ERα and ERβ. n=3. (D) FSH decreased *Npr2* mRNA levels in COCs after a culture of 24 h, even when E2 was added. n=3.

Data represent the mean ± SEM. FSH, 0.1 IU/ml; LH, 1.0 μg/ml; E2, 0.1 μM; Con., Control. Different letters (a-c) indicate significant differences between groups ($p < 0.05$, ANOVA and Holm–Šidák test) in profiles in (C-E).

Figure S1

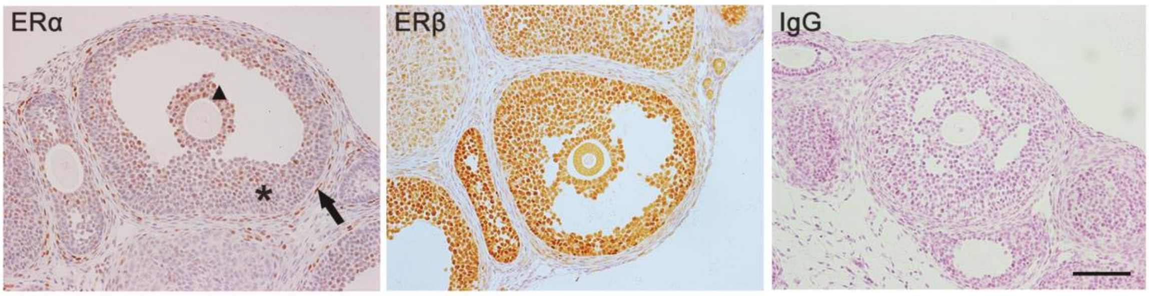

Figure S2

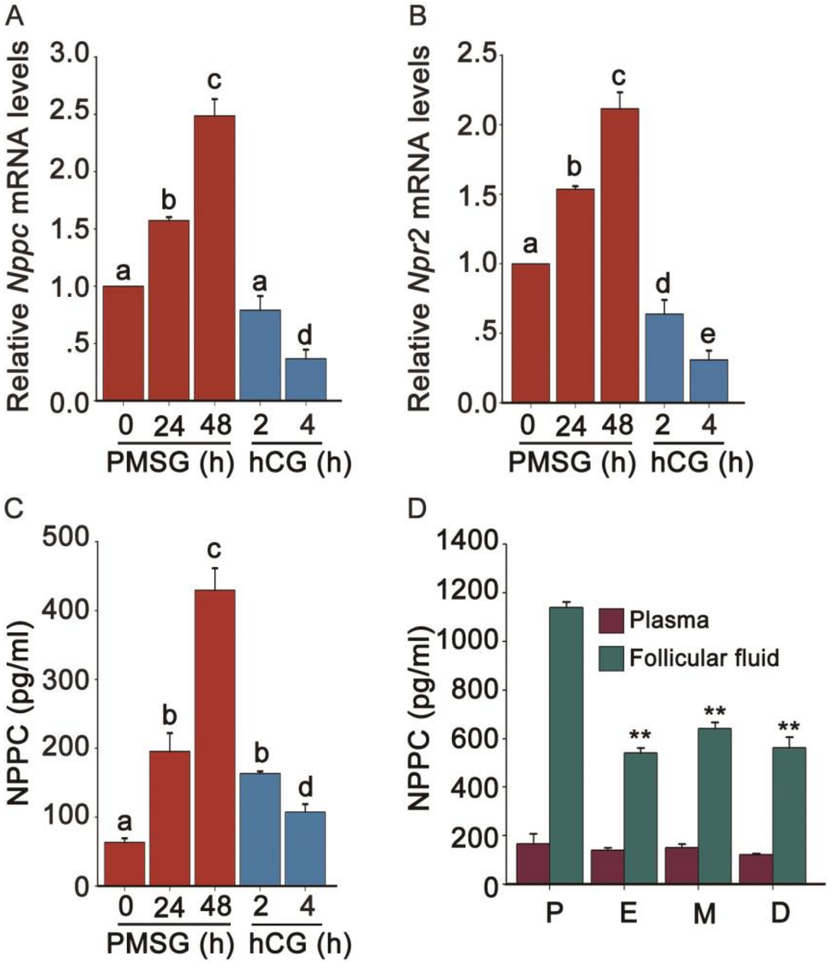

A
Relative Nppc mRNA levels
3.0
2.5
2.0
1.5
1.0
.5
0.0
a
b
c
a
d
0
24
48
2
4
PMSG (h)
hCG (h)
B
Relative Npr2 mRNA levels
2.5
2.0
1.5
1.0
.5
0.0
a
b
c
d
e
0
24
48
2
4
PMSG (h)
hCG (h)
C
NPPC (pg/ml)
500
400
300
200
100
0
a
b
c
b
d
0
24
48
2
4
PMSG (h)
hCG (h)
D
NPPC (pg/ml)
1400
1200
1000
800
600
400
200
0
Plasma
Follicular fluid
**
**
**
P
E
M
D

Figure S3

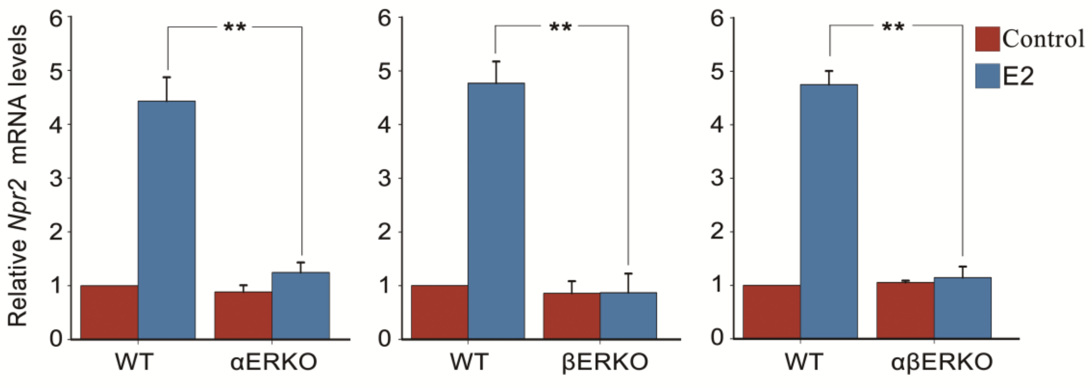

Relative *Npr2* mRNA levels
6
5
4
3
2
1
0
**
WT
αERKO
βERKO
αβERKO
Control
E2

Figure S4

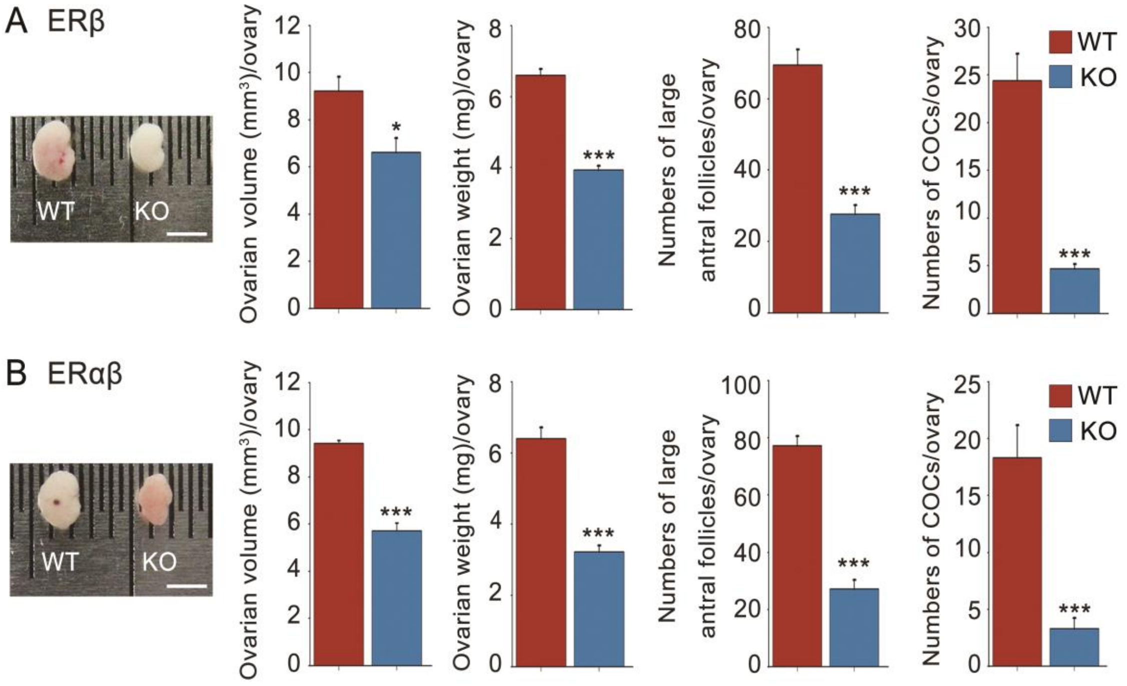
A ERβ
WT
KO
Ovarian volume (mm³)/ovary
Ovarian weight (mg)/ovary
Numbers of large antral follicles/ovary
Numbers of COCs/ovary
*
***
B ERαβ
WT
KO

Figure S5

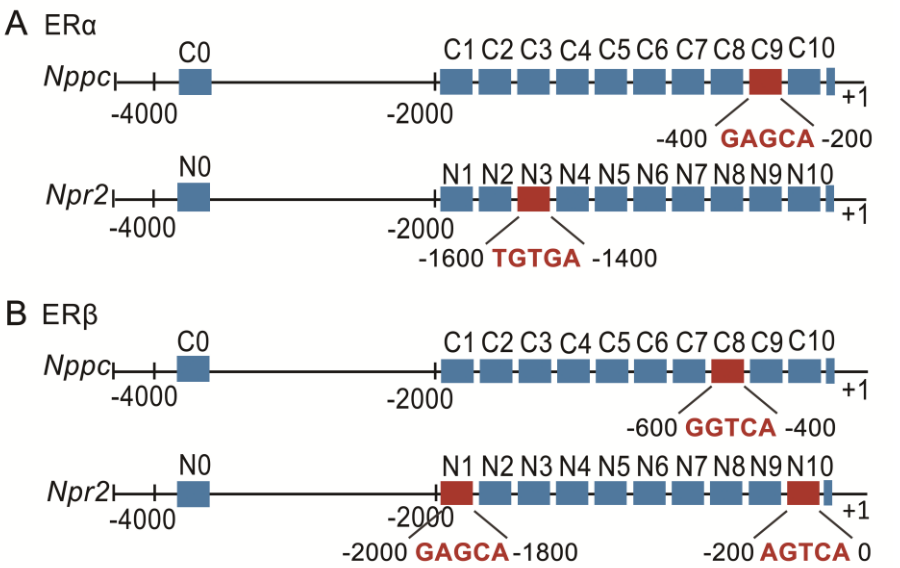
A ERα
Nppc
C0
C1 C2 C3 C4 C5 C6 C7 C8 C9 C10
-4000
-2000
+1
-400 GAGCA -200
Npr2
N0
N1 N2 N3 N4 N5 N6 N7 N8 N9 N10
-4000
-2000
+1
-1600 TGTGA -1400
B ERβ
Nppc
C0
C1 C2 C3 C4 C5 C6 C7 C8 C9 C10
-4000
-2000
+1
-600 GGTCA -400
Npr2
N0
N1 N2 N3 N4 N5 N6 N7 N8 N9 N10
-4000
-2000
+1
-2000 GAGCA -1800
-200 AGTCA 0

Figure S6

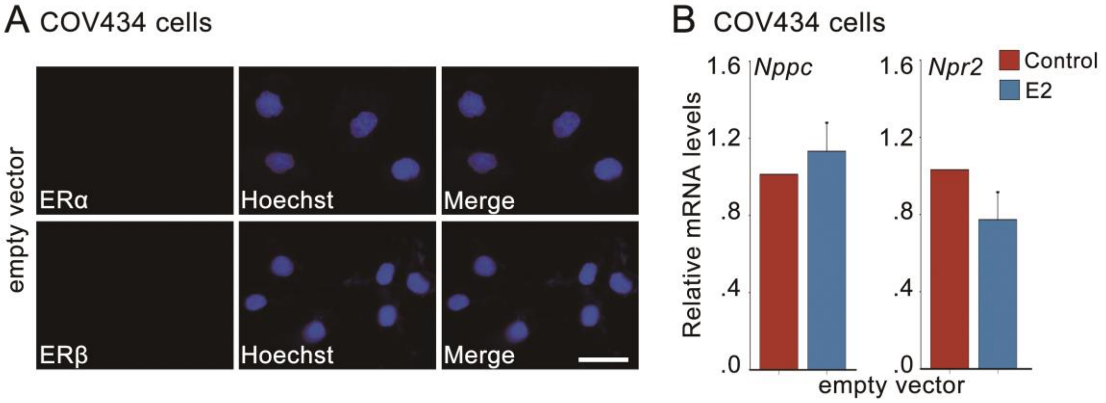

A COV434 cells
empty vector
ERα
Hoechst
Merge
ERβ
Hoechst
Merge
B COV434 cells
Relative mRNA levels
1.6
1.2
.8
.4
.0
Nppc
Npr2
Control
E2
empty vector

Figure S7

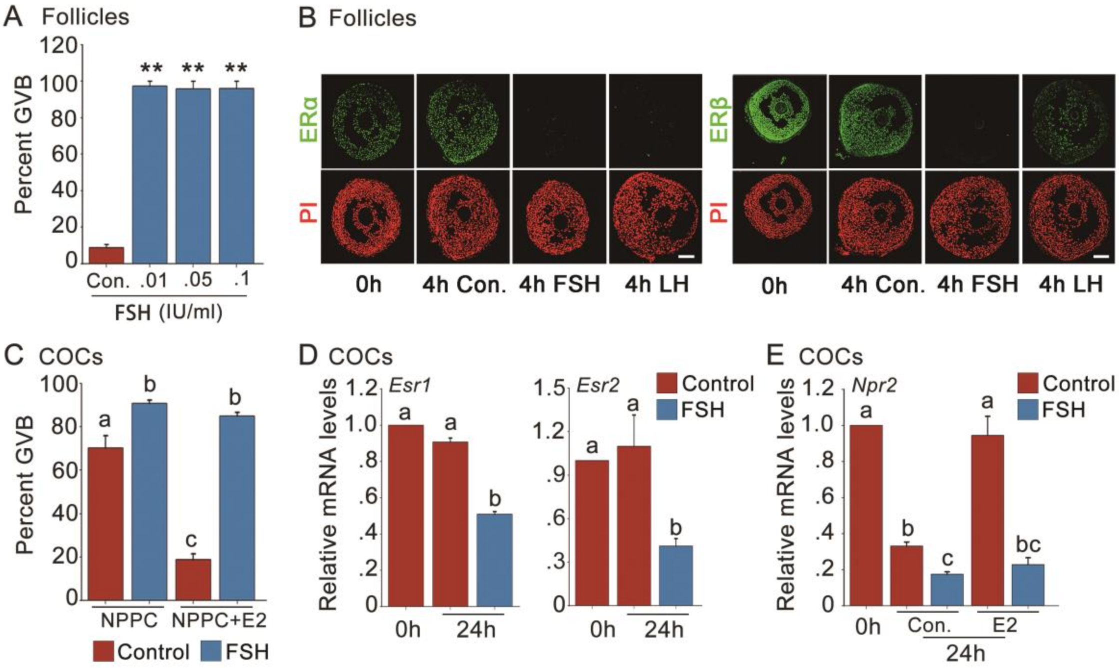
A Follicles
Percent GVB
120
100
80
60
40
20
0
**
Con.
.01
.05
.1
FSH (IU/ml)
B Follicles
ERα
PI
ERβ
0h
4h Con.
4h FSH
4h LH
C COCs
Percent GVB
a
b
c
NPPC
NPPC+E2
Control
FSH
D COCs
Relative mRNA levels
Esr1
Esr2
0h
24h
E COCs
Npr2
bc
Con.
E2
24h

Supplementary Tables

Table S1: Primers for RT-qPCR.

Genes	Forward primer (5'-3')	Reverse primer (5'-3')
Nppc-Mus	GGTCTGGGATGTTAGTGCAGCTA	TAAAAGCCACATTGCGTTGGA
Npr2-Mus	GCTGACCCGGCAAGTTCTGT	ACAATACTCGGTGACAATGCAGAT
Esr1-Mus	AAAGGCGGCATACGGAAAGAC	CTCCTGAAGCACCCATTTCAT
Esr2-Mus	CTGTGCCTCTTCTCACAAGGA	TGCTCCAAGGGTAGGATGGAC
Gapdh-Mus	GGTGAAGGTCGGTGTGAACG	CTCGCTCCTGGAAGATGGTG
Nppc-Homo	GCAAATACAAAGGAGCCAACAAG	CATGGAGCCGATTCGGTCC
Npr2-Homo	TGACCCCGACCTGCTGTTA	CGAACCAGGGTACGATAATGG
Esr1-Homo	CCCACTCAACAGCGTGTCTC	CGTCGATTATCTGAATTTGGCCT
Esr2-Homo	AGCACGGCTCCATATACATACC	TGGACCACTAAAGGAGAAAGGT
β-actin-Homo	CTCACCATGGATGATGATATCGC	AGGAATCCTTCTGACCCATGC

Table S2: Primers for plasmid construction.

Genes	Forward primer (5'-3')	Reverse primer (5'-3')
Nppc-Mus	TGAGTCATTTCCCAAACGAAAGGCTG	AGTGCACCGATGTAGCATAGATGACTT
Npr2-1-Mus	ACTTCTCTTCCTGGCCCTCTTC	AAGCAGCCCGAGCTGTCCAATTG
Npr2-2-Mus	GAATGTGTTTATGTGCATGTCC	TCAGCACCCCGGGCCTCTA
Esr1-Mus	ATGACCATGACCCTTCACACC	GATCGTGTTGGGGAAGCCCTCTG
Esr2-Mus	ATGTCCATCTGTGCCTCTTCTCACA	CTGTGACTGGAGGTTCTGGGAGCC
Esr1-Homo	CGCGGATCCATGACCATGACCCTCCACACCA	CCGCTCGAG
Esr2-Homo	CGCGGATCCATGGATATAAAAAACTCACCA	CCGCTCGAGTCACTGCTCCATCGTTGCTTC

Table S3: Primers for the ChIP assay.

Genes	Forward primer (5'-3')	Reverse primer (5'-3')
Nppc-C0	GCCAGCAGCTCCTGCCTACC	CCTTTCCAAGAAGGAGATGG
Nppc-C1	TCATTTCCCTCGCTCAAGCCT	GCTATAGGGACCGGGCCACT
Nppc-C2	CAGAAGGCAACTACAACCCCA	TGGGAGGTTAGGAGGCAGGTA
Nppc-C3	GCCTCCTAACCTCCCAACAC	GCAGAGGATGGGGTTAGTAGA
Nppc-C4	CGCCCCCATGTTTGAGCGTG	CTCCTGATACGTGTCTGTAC
Nppc-C5	GCCTCCTAACCTCCCAACAC	GCCACGGGGGCTCCCTCTTC
Nppc-C6	CCTCCGGGCCGTCGATTCGG	AGCAGGATTGCCAAGCGAGC
Nppc-C7	CAATCCTGCTCCGCATCCGCC	CGCGACAGCACCCACCTTCGGT
Nppc-C8	TGTCGCGGGGACGCTGGGCT	GCAGGTCATGCTGGGCACATT
Nppc-C9	GGCACGGGAAGAGCAATGGG	CTGTCGGAGAAAAGAGTGGA
Nppc-C10	GTCCCGAGAACCCCGCCAGG	CTCGTGCAGAAGGCGGGCCC
Npr2-N0	ACGGAATTCCCCAAGCTCTGC	ACTGGATAGCCCAGAGACCA
Npr2-N1	CTCCAGATAAGGGGTACCTGGA	TATTGGATCCATCAGCCATCTG
Npr2-N2	GGCATAAGAGCAGAACGACCGT	TTCCTGGGTAGGGAAAACTCTA
Npr2-N3	TAAATGGATACCCAGTGATTG	CATACTGTAAGGGCCTTCCTCT
Npr2-N4	TACAGTATGGGGACGCCCATGA	TGTGACTTCCCCGGCAGGCC
Npr2-N5	CTCGGCCGCCGCCGCGGGCGCA	CATCCGGAGCTGCGGCGGCC
Npr2-N6	GGACCAGCGCCCGGGCCCGTT	CAAAGAGCTGCGAGAAGCCAG
Npr2-N7	CTCTCCCGGCCCGATCAGCTGT	ATAGGGTGGCAGAAGGAAGA
Npr2-N8	CCTTAGTCCCTGGACCTGGCT	CTCTGGCCTACTGGGCAGGCA
Npr2-N9	CGGCCCCTGGTTCGGGGGACC	GTGCCTCCACAGCCAGTGCCA
Npr2-N10	GGCTGTGGAGGCACTGGGCC	GAGGGGAAGGCGCCAGTGTGA

Supplementary References

1. Murr SM, Geschwind, II, Bradford GE. Plasma LH and FSH during different oestrous cycle conditions in mice. *J Reprod Fertil* 1973, **32**(2)**:** 221-230.

2. Pedersen T, Peters H. Proposal for a classification of oocytes and follicles in the mouse ovary. *J Reprod Fertil* 1968, **17**(3)**:** 555-557.

3. John GB, Shidler MJ, Besmer P, Castrillon DH. Kit signaling via PI3K promotes ovarian follicle maturation but is dispensable for primordial follicle activation. *Dev Biol* 2009, **331**(2)**:** 292-299.

4. Chen C, Cai Q, He W, Li Z, Zhou F, Liu Z*, et al.* An NKX3.1 binding site polymorphism in the l-plastin promoter leads to differential gene expression in human prostate cancer. *International Journal of Cancer* 2016, **138**(1)**:** 74-86.
